# Buffalo Obstetrical Society

**Published:** 1890-07

**Authors:** Wm. C. Krauss

**Affiliations:** Secretary


### BUFFALO OBSTETRICAL SOCIETY.

Reported by WM. C. KRAUSS, M. D., Secretary.

STATED meeting held May 27, 1890.

The president, Dr. C. C. FREDERICK, in the chair.

The essayist of the evening, Dr. WILLIAM H. THORNTON, prepared the following paper, which was read by the secretary, entitled

### LABOR PAINS AND AFTER PAINS.

In the management of obstetric cases our duty is by no means all done when, having assured ourselves that the presentation is normal, we leave the case to nature until the completion of the second stage. I believe we should pay more attention than is usually done to the relief of suffering in these cases, and endeavour to guard against the exhaustion of the muscular and nervous systems following a prolonged labor. If we should conduct cases on the basis of giving patients as much freedom from pain as is consistent with their safety and a full regard for after effects, we would not only do what is humane, but we would in many instances bring our patients through to a more speedy and better convalescence, and avoid numerous of the preventable injuries of parturition.

My object in bringing this subject before the society is to discuss and elicit discussion of the various ways and means of alleviating and shortening the suffering incidental to an ordinary case of childbirth, and to consider the extent to which we should apply these means. These cases form so important an element of our practice that I feel justified in taking your attention this evening for a consideration of what I regard as a very important element in their management. I do this with the hope, not so much of suggesting things new, as of bringing freshly and consecutively to our minds the various therapeutical and mechanical resources of our art applicable to the relief of the suffering of womankind in this time, which to her is usually the culmination of months of anxiety.

The pains in the beginning of labor are chiefly due to the pressure on nerve filaments caused by contraction of the muscular fibres to which they are distributed, and are shortly followed by the severer ones due to the stretching and dilatation of the cervix. This is accomplished partly by muscular contraction, and partly by mechanical pressure of the soft fluid wedge formed by the projecting bag of waters or the presenting part. As this process goes on, the cervix gradually grows thinner and shorter, and is finally taken up to form with the body of the womb and the vagina a continuous canal. In the second stage the presenting part, passing into the vagina, presses upon the vaginal nerves and the large plexuses lying within the pelvis. Then, descending still lower, it presses upon the bladder and rectum, and finally stretches open the vulva and perineum.

The pains preceding the dilatation of the cervix usually come with considerable intervals between each, and are not of a very severe character. But in many cases the pains caused by the opening of the cervix, although not so severe as those of the later stages, are of such a character that they are more exasperating and wearing to the patient than those later on, when she can appreciate the results which they accomplish. This is especially true when we have a rigid cervix. This may be at times thin and hard, or thick and unyielding. At times the induration is due to cicatrices resulting from former labors. Unless this condition is relieved, patients exhaust their strength and courage and accomplish little toward the progress of the labor. Formerly this condition of rigid cervix was treated by venesection, or by minute doses of tartar emetic. At present better methods have thrown these old, but usually efficient, methods into disuse. In overcoming this difficulty it is important to do so with a remedy which will give relief, without at the same time retarding the labor. In France a warm bath is much used — either an entire bath or a hip bath. A midwife often attempts to secure the same result by seating the patient over a pail of steaming hot water. Playfair speaks highly of a douche of tepid water applied to the cervix. Either the douche or the bath are, however, so inconvenient to use that they would be apt to be postponed as long as possible. The application of extract of belladonna has been advised, but is not shown to be of much value.

Stretching the cervix with the fingers or with rubber dilators is sometimes useful, especially when the early escape of the waters has thrown this work upon the hard presenting part instead of the usual fluid wedge. Chloroform may be administered to the extent of producing muscular relaxation, but there are many objections to using it at this stage of labor to that extent. When cocaine first came into use great things were expected of it here, but it has usually disappointed those who have tried it. In chloral, however, we have a drug which in nearly all cases will produce the desired results, without being subject to the objections that apply to the other methods suggested. It acts here in a double way,—by relaxing the tense and hardened muscle, and by producing a drowsy condition in which the patient feels the pain much less acutely. Dr. Taylor, of Cincinnati, made some experiments with antipyrin, but found that though it relieved the pain to some extent, it had some depressing effect and retarded the labor. I am not certain, however, that his experiments were carried out to a sufficient extent to be conclusive as regards the utility of this agent. Chloral, to be effective here, should usually be given in doses of about fifteen grains, and repeated two or three times at intervals of fifteen or twenty minutes; or, as has been recommended by some, in a dose of one dram by enema, and repeated in four or five hours, if necessary. Given in this way, chloral not only does not lessen the force of the contractions but seems rather to make them regular, although it does relieve largely the severity of suffering.

Charpentier speaks in high terms of the use of chloral. He says: "We have seen that the partisans of modern anesthesia resort in particular to chloroform during the stage of expulsion, but this period, although perhaps the most painful, is not the most unendurable. It is then that parturients take account of the progress of their labor, regain courage, listen readily to the counsel of their physicians, and are in reality less irritable and excitable, except, perhaps, during the last contractions, than during the first stage of labor." Now, it is during the first stage of labor, that is to say the period of dilatation, that chloral has appeared to me of incontestable utility. When the pains in the back are very pronounced or dilatation is slow, either because of feeble or irregular pains or because of rigidity of the cervix, in primipara, for example, then it is that I have used chloral. The drug has never seemed to me to have a bad effect upon the pains. In the second stage of labor, the expulsory stage, the action of chloral has seemed to me less marked. We then have recourse to chloroform, even as when manual or instrumental assistance is called for.

In the second stage, if the pains are very severe and progress is slow, anesthetics are often called for. Given carefully and intermittently, in proper cases, the good results from their use certainly outweigh the occasional objections to them. Although at times they retard the labor, it very often happens that labor is greatly facilitated by their use, partly by relaxing the vaginal and perineal muscles, and partly by lessening the suffering, so that the patients will bear down longer and harder than when the pain is felt more acutely. If the anesthetic is given at the beginning of the pain and discontinued at its expiration, the patient will experience great relief therefrom, no delay will be caused to the labor, and there will be freedom from the occasional unpleasant after-effects produced by full narcosis. Again I will quote from Charpentier, for I fully agree with most that he says on this subject. He separates the administration of chloroform into what he calls the surgical and the obstetrical dose. Given in the obstetrical dose he says:

(1). Chloroform given in small doses unquestionably takes the edge off the pains, and thus gives the parturient both moral and physical strength. At the same time the intervals between the contractions are lengthened. (2). Complete analgesia we have never observed, except under deep anesthesia, and then the drug is administered in a surgical dose. (3). At times chloroform, instead of quieting, excites the patient to such a degree that we are obliged to cease its administration. (4). Occasionally it has seemed to us that chloroform has diminished the contractile powers of the uterus, and therefore caused hemorrhage more or less grave. This can easily be guarded against by the administration of a dose of ergot immediately after the completion of the third stage. (5). Its action on the fetus is nil. (6). Administered during the period of expulsion, chloroform certainly diminishes pain, but its effect on the contractions of the abdominal muscles and the resistance of the perineum has seemed to us less marked than is generally supposed. There is another anesthetic to which we often have recourse, and which has frequently rendered us yeoman service. We refer to chloral."

Ether can be used in cases where chloroform is inadmissable, but owing to the longer time requisite for its administration and the inability to administer it intermittingly as easily as chloroform, the latter should be preferred.

We next come to a consideration of the mechanical means at our command to shorten labor.

Dr. Opie, of Baltimore, favors the use of manual pressure over the fundus, and quotes Kristeller, of Berlin, who claims for it the following advantages:

(1). It shortens the duration of labor. (2). The normal position of the child is preserved. (3). The application of forceps is frequently rendered unnecessary. (4.) It thereby aids in protecting the perineum. (5.) It facilitates and hastens the nevertheless often necessary forceps delivery. (6.) It preserves the upward extension of the arms in breech delivery. (7.) It hastens the expulsion of the shoulders after the birth of the head. It has been said that the results are more favorable than in forceps cases; that there is less local inflammation and less septicemia."

This method of assistance is not, I think, much used in this country, but it doubtless in some cases would be of decided advantage.

There is a wide diversity in the practice of different physicians as to the class of cases in which they would apply the forceps, some maintaining that it should never be used when the forces of nature are equal to the task of delivery, while others maintain that this is an unnecessary and cruel restriction of the use of this humanitarian aid.

As this paper is written simply to discuss the methods and extent to which we should take part in an ordinary case of uncomplicated labor, I shall speak of the application of the forceps only in the second stage, since in the first stage its application is a matter of such difficulty and danger that its use should be decided for or against solely on the ground of necessity; but in the second stage other elements enter into the consideration. The alliterative term, "meddlesome midwifery," is much used by those who are accustomed to speak strongly against the use of forceps except in cases of absolute necessity. The question then comes up at what point does our instrumental aid become meddlesome? Are there not many cases in which nature alone can complete the case in the course of several hours, but where we are fully justified in giving instrumental aid to greatly reduce this period? I believe that where we have in such cases patients of delicate physique, of a highly nervous temperament, or those who bear pain very badly, or where the pains are regular but weak and inefficient, the amount of nervous and physical exhaustion which we save them more than justifies us in giving instrumental aid. I shall not discuss the effect of this aid upon the maternal structures, for I assume that in cases where such results are likely to follow, the use of forceps will be postponed as long as is consistent with the well-being of the patient in other ways. But we should bear in mind that a slough from long-continued pressure is as bad, or worse, for the patient than a laceration due to instrumental delivery. We would all, I am sure, condemn most strongly the use of the forceps as a matter of convenience to the accoucheur in saving time.

If our patients were all in a natural, normal condition, there would be much more force to the arguments urged against interfering with nature in the performance of a physiological function; but we must treat our cases as they are, and make allowances for departures from the normal standard of energy and physical and nervous strength.

There is a natural tendency to become somewhat indifferent to the suffering of our patients, and not give to that element the weight to which it is entitled in deciding upon our management of an obstetric case.

The third stage of labor being past, we should direct our attention to the prevention of after pains, which, in some cases, give rise to as much suffering, or at least annoyance, to the patient as the pains of delivery. This can only be done at the expense of some time and attention by the physician. The placenta being delivered, we should at once, by gentle and persistent manipulation, having emptied the womb of all clots, bring it into a condition of firm and uniform contraction. It is then my habit, in most cases, to administer half a dram of Squibb's ergot, and direct that it be repeated in case the after pains are severe or the flow should become too great. By this means we have an assurance, which we can get in no other way, that the uterus will not relax soon after our departure and put our patients in extreme danger.

A neatly applied abdominal bandage also aids, by making slight pressure, in keeping up uterine contraction, and it certainly gives the patient an agreeable feeling of support. For the relief of such pains as are not prevented by the means suggested, it has been my custom for some time to order five grain doses of acetanilid, repeated in three to six hours, as occasion requires. This has given very satisfactory results. Chloral also is of much benefit here.

In thus urging, as I have, that greater attention should be paid to the question of pain in labor cases, I by no means wish to be understood as advocating the wholesale use of anesthetics and forceps; but I do wish to maintain that more consideration should be given to this subject, and that the use or non-use of anesthetics and other aid be not always decided upon the ground of necessity, but that in cases of prolonged and extreme pain these agents be given more than a potential place, and that as a matter of humanity some of them be used unless there are positive indications to the contrary.

DISCUSSION.

Dr. W. S. Tremaine.—The custom among the Mexican women during labor is to sit in their husband's laps and have them apply force to the abdomen. This generally accelerates labor, and is analagous to a procedure sometimes applied during protracted labor.

Dr. Delancey Rochester.—I do not think it is advisable to give ergot for the after pains. If the uterus is thoroughly emptied of all clots there will be no after pains. Ergot and opium are better than ergot alone. I have given antipyrin in several cases without any results.

Dr. A. E. Persons.—I think that in some cases we may use means to relieve pains. I do not believe in pushing chloroform very far, except only during the application of forceps. I usually permit the patient to administer the chloroform herself; then there will be no danger of an overdose, and you will avoid complete anesthesia. Chloral, per rectum, is also a very good method.

Dr. Marcel Hartwig.—Hydrate of chloral does not diminish labor pains at all. It increases the pains and contractions, but does not relieve the pain. How it acts per rectum I cannot understand, as it is irritating and apt to be expelled. Cocaine I have not used. Hydrobromate of hyoscine might be of value in these cases. Ergot seems to relieve the after pains.

Dr. A. H. Briggs.—It has been my experience and observation that between drugs and operative measures I have always chosen the latter, viz., fingers and forceps. I had a case years ago where a patient had dropsy, and the urine was almost all albumen. Dr. Rochester advised to induce labor. In the afternoon I used simply my index finger, dilating the os; the pains began and labor terminated three hours afterward. In the administration of ergot I never give it before the birth of the child. In the use of instruments I don't follow the first advice given me, but use them early, when there is the slightest tendency to delay.

Dr. P. W. Van Peyma.—As to chloral, I formerly used it considerably, but have now given it up and use morphia and atropia in the first stage. As to steaming, I don't object to it nor do I second it; as to chloroform, I seldom give it in the first stage, but do in the second stage, and put the patient beyond the exciting stage, so as to quiet the feeling of pain. With forceps, the simple introduction of the blades will sometimes cause the head to descend. I have also used the fingers to dilate the os. One can start the labor pains artificially. Hypnotism has also been resorted to to ameliorate the pains of labor.

Dr. Thos. Lothrop.—I don't believe it possible to ever overcome the pains of labor; it is but the expression of a natural law. I don't give any drugs, and don't interfere in normal cases. As women advance in the scale of civilization, deliverances, become more difficult. In case the pains are biting, irritable and exhausting, I give morphia ¼ – ⅓ of a grain. Dr. White's remedy was Dover's powder, and I think it was good practice. In old primiparæ, with rigidity of the soft parts, nothing is so efficacious as hot water. It does not mitigate the pains, but helps dilate the soft parts. In cases of rigid os nothing compares with venesection. In the latter part of the second stage I occasionally give chloroform.

Dr. C. C. Frederick.—For the more acute pains of the second stage chloroform has given good results in my hands. In cases of rigid os, a combination of chloral and morphia act better in hastening the first stage than either alone.

Dr. Wm. C. Krauss.—In the woman's hospital at Munich ergot is given only after the birth of the child. It is never resorted to beforehand. I have never seen any anesthetic administered to alleviate the pains of the second stage, perhaps because the patients under observation were city cases. If a patient is susceptible and has been hypnotized several times beforehand, this may be resorted to during the second stage, but I don't believe it would be good practice as a rule.

Dr. R. L. Banta.—In the use of morphia for mitigating the pains of the first stage, it acts well and helps to dilate the os. I think we ought to mitigate the pains of labor when we are able to do so.

Dr. H. C. Buswell.—In the pains of labor I usually give ½ - ¾ of a grain of morphia.

